# Strongly singular integrals along curves on *α*-modulation spaces

**DOI:** 10.1186/s13660-017-1458-0

**Published:** 2017-08-09

**Authors:** Xiaomei Wu, Xiao Yu

**Affiliations:** 1 Xingzhi College, Zhejiang Normal University, Jinhua, 321004 P.R. China; 2 Department of Mathematics, Shangrao Normal University, Shangrao, 334001 P.R. China

**Keywords:** *α*-modulation spaces, strongly singular integrals, Besov spaces, homogeneous curves

## Abstract

In this paper, we study the strongly singular integrals

$$T_{n, \beta, \gamma}f(x)=\mathrm{p.v.} \int_{-1}^{1}f\bigl(x-\Gamma_{\theta}(t) \bigr)\frac {e^{-2\pi i \vert t \vert ^{-\beta}}}{t \vert t \vert ^{\gamma}}\,dt $$

along homogeneous curves $\Gamma_{\theta}(t)$. We prove that $T_{n, \beta, \gamma}$ is bounded on the *α*-modulation spaces, including the inhomogeneous Besov spaces and the classical modulation spaces.

## Introduction

The two dimension strongly singular integrals along curves $T_{\beta, \gamma}$ are defined by

$$T_{\beta, \gamma}(f) (x, y)=\mathrm{p.v.} \int_{-1}^{1}f\bigl(x-t,y-\Gamma(t)\bigr) \frac {e^{-2\pi i \vert t \vert ^{-\beta}}}{t \vert t \vert ^{\gamma}}\,dt, $$

where $x, y\in\mathbb{R}, \beta, \gamma>0$. Zielinski [1] showed that $T_{\beta, \gamma}$ is bounded on $L^{2}(\mathbb{R}^{2})$ along the curve $(t, t^{2})$ if and only if $\beta >3\gamma$. Later, Chandarana [2] extended the result to the general curves $(t, \vert t \vert ^{m})$ or $(t, \operatorname{sgn}(t) \vert t \vert ^{m})$ with $m\geq2$ and showed that $T_{\beta, \gamma}$ is bounded on $L^{p}(\mathbb{R}^{2})$ for

$$1+\frac{3\gamma(\beta+1)}{\beta(\beta+1)+\beta-3\gamma}< p< \frac{\beta (\beta+1)+(\beta-3\gamma)}{3\gamma(\beta+1)}+1. $$

Moreover, Chandarana also studied the strongly singular integrals along curves in $\mathbb{R}^{3}$ (see [2] for details). In [3], Chen *et al.* considered the operator for high dimension *n*. Let $\theta=(\theta_{1}, \theta_{2}, \ldots, \theta_{n})\in\mathbb{R}^{n}$, and

$$\Gamma_{\theta}(t)=\bigl(\theta_{1} \vert t \vert ^{p_{1}}, \theta_{2} \vert t \vert ^{p_{2}}, \ldots, \theta _{n} \vert t \vert ^{p_{n}}\bigr) $$

or

$$\Gamma_{\theta}(t)=\operatorname{sgn}(t) \bigl(\theta_{1} \vert t \vert ^{p_{1}}, \theta_{2} \vert t \vert ^{p_{2}}, \ldots, \theta_{n} \vert t \vert ^{p_{n}}\bigr). $$

Then the operator $T_{n, \beta, \gamma}$ is defined as

$$T_{n, \beta, \gamma}f(x)=\mathrm{p.v.} \int_{-1}^{1}f\bigl(x-\Gamma_{\theta}(t) \bigr)\frac {e^{-2\pi i \vert t \vert ^{-\beta}}}{t \vert t \vert ^{\gamma}}\,dt\quad \bigl(\beta>\gamma>0, x\in\mathbb{R}^{n}\bigr). $$

Suppose $p_{1}, p_{2}, \ldots, p_{n}, \alpha$ and *β* are positive numbers. In [3], the authors proved that $T_{n, \beta, \gamma}$ is bounded on $L^{p}(\mathbb{R}^{n})$ whenever $\beta>(n+1)\gamma$ and

$$\frac{2\beta}{2\beta-(n+1)\gamma}< p< \frac{2\beta}{(n+1)\gamma}. $$

Later, Cheng-Zhang [4] and Cheng [5] extended the results to the modulation space. They showed that the strongly singular integral $T_{n, \beta, \gamma}$ is bounded on the modulation spaces $M_{p,q}^{s}$ for all $p>0$. It is worth to point out that the modulation space is a better substitution to study the strongly singular integrals because there is no restriction on the index *p*.

Here we will consider the strongly singular integrals along homogeneous curves $T_{n, \beta, \gamma}$ on the *α*-modulation spaces. The *α*-modulation spaces $M_{p,q}^{s,\alpha}$ were first introduced by Gröbner in [6]. They contain the inhomogeneous Besov spaces $B_{p,q}^{s}$ in the limit case $\alpha=1$ and the classical modulation spaces $M_{p,q}^{s}$ in the case $\alpha =0$, respectively. It is proposed as an intermediate function space; see [6, 7] for more details.

In recent years, there were numerous papers on these spaces and its applications, such as [7–13] and the references therein. Motivated by the work of Cheng-Zhang [4] on the modulation spaces, one naturally expects that the strongly singular integral operators $T_{n, \beta, \gamma}$ have the boundedness property on the *α*-modulation spaces for all $0\leq\alpha\leq1$. In this paper, we will affirm this.

This paper is organized as follows. In Section 2, we will recall the definition of the *α*-modulation spaces and the Besov spaces. Some lemmas will also be presented in this section. In Section 3, we will give the main results and prove the theorems. In addition, we will consider the strongly singular integrals along a well-curved $\Gamma (t)$ in $\mathbb{R}^{n}$. Throughout this paper, we use the notation $A\preceq B $ meaning that there is a positive constant C independent of all essential variables such that $A\leq CB$. We denote $A\sim B$ to stand for $A\preceq B $ and $B\preceq A$.

## Preliminaries and lemmas

Before giving the definition of the *α*-modulation spaces, we introduce some notations frequently used in this paper. Let $\mathcal {S}=\mathcal{S}(\mathbb{R}^{n})$ be the subspace of $C^{\infty}(\mathbb {R}^{n})$ of Schwartz rapidly decreasing functions and $\mathcal {S}'=\mathcal{S}'(\mathbb{R}^{n})$ be the space of all tempered distribution on $\mathbb{R}^{n}$. For $k=(k_{1}, k_{2}, \ldots, k_{n})\in \mathbb{Z}^{n}$, we denote

$$\vert k \vert =\bigl(k_{1}^{2}+k_{2}^{2}+ \cdots+k_{n}^{2}\bigr)^{\frac{1}{2}}, \qquad\langle k\rangle = \bigl(1+ \vert k \vert ^{2}\bigr)^{\frac{1}{2}}. $$

We define the ball

$$B_{k}^{r}:=\bigl\{ \xi\in\mathbb{R}^{n}: \bigl\vert \xi-\langle k\rangle^{\frac{\alpha }{1-\alpha}}k \bigr\vert < r\langle k \rangle^{\frac{\alpha}{1-\alpha}}\bigr\} $$

and $B_{k}^{2r}$ denotes

$$\bigl\{ \xi\in\mathbb{R}^{n}: \bigl\vert \xi-\langle k \rangle^{\frac{\alpha}{1-\alpha }}k \bigr\vert < 2r\langle k\rangle^{\frac{\alpha}{1-\alpha}}\bigr\} . $$

The Fourier transform $\mathcal{F}(f)$ and the inverse Fourier transform $\mathcal{F}^{-1}(f)$ are defined by

$$\begin{aligned} &\mathcal{F}(f) (\xi)=\hat{f}(\xi)= \int_{\mathbb{R}^{n}}f(x)e^{-2\pi i x\cdot\xi}\,dx, \\ &\mathcal{F}^{-1}(f) (\xi)=\breve{f}(\xi)= \int_{\mathbb {R}^{n}}f(x)e^{2\pi i x\cdot\xi}\,dx. \end{aligned}$$

To define the *α*-modulation spaces, we introduce the *α*-decomposition. Let *ρ* be a nonnegative smooth radial bump function supported in $B(0,2)$, satisfying $\rho(\xi)=1$ for $\vert \xi \vert <1$ and $\rho(\xi)=0$ for $\vert \xi \vert \geq2$. For any $k=(k_{1}, k_{2}, \ldots, k_{n})\in\mathbb{Z}^{n}$, we set

$$\rho_{k}^{\alpha}(\xi)=\rho \biggl(\frac{\xi-\langle k\rangle^{\frac{\alpha }{1-\alpha}}k}{r\langle k\rangle^{\frac{\alpha}{1-\alpha}}} \biggr) $$

and denote

$$\eta_{k}^{\alpha}(\xi)=\rho_{k}^{\alpha}(\xi) \biggl(\sum_{l\in\mathbb {Z}^{n}}\rho_{l}^{\alpha}( \xi) \biggr)^{-1}. $$

It is easy to check that $\{\eta_{k}^{\alpha}\}_{k\in\mathbb{Z}^{n} }$ satisfy

2.1 $$\begin{aligned} &\operatorname{supp}\eta_{k}^{\alpha}\subset B_{k}^{2r}; \end{aligned}$$

2.2 $$\begin{aligned} &\eta_{k}^{\alpha}(\xi)=c, \quad \forall\xi\in B_{k}^{r}; \end{aligned}$$

2.3 $$\begin{aligned} &\sum_{k\in\mathbb{Z}^{n}}\eta_{k}^{\alpha}(\xi) \equiv1,\quad \forall\xi\in \mathbb{R}^{n} \end{aligned}$$

and

2.4 $$ \bigl\Vert \mathcal{F}^{-1}\eta_{k}^{\alpha} \bigr\Vert _{L^{1}}\preceq1. $$

Corresponding to the above sequence $\{\eta_{k}^{\alpha}\}_{k\in\mathbb {Z}^{n} }$, we can construct an operator sequence $\{\Box_{k}^{\alpha}\} _{k\in\mathbb{Z}^{n} }$ by

$$\Box_{k}^{\alpha}=\mathcal{F}^{-1} \eta_{k}^{\alpha}\mathcal{F}. $$

For $0\leq\alpha<1, 0<p, q\leq\infty, s\in\mathbb{R}$, using this decomposition, we define the *α*-modulation spaces as

$$M_{p,q}^{s,\alpha}\bigl(\mathbb{R}^{n}\bigr):= \biggl\{ f \in\mathcal{S}': \Vert f \Vert _{M_{p,q}^{s,\alpha}}= \biggl(\sum _{k\in\mathbb{Z}^{n}}\langle k\rangle ^{\frac{sq}{1-\alpha}} \bigl\Vert \Box_{k}^{\alpha}f \bigr\Vert _{L^{p}}^{q} \biggr)^{\frac {1}{q}}< \infty \biggr\} . $$

We have the usual modification when $p, q=\infty$. We denote $M_{p,q}^{s,0}=M_{p,q}^{s}$. It is the classical modulation space. Its related decomposition is called uniform decomposition; see [6, 7] and [14] for details. In order to define the Besov spaces, we introduce the dyadic decomposition. Let *ψ* be a smooth bump function supported in the ball $\{\xi: \vert \xi \vert \leq\frac{3}{2}\}$. We may assume $\psi(\xi)=1$ if $\vert \xi \vert \leq\frac{4}{3}$. Denote $\phi(\xi )=\psi(\xi)-\psi(2\xi)$ and a function sequence $\{\phi_{j}\}_{j=0}^{\infty}$:

$$\textstyle\begin{cases} \phi_{j}(\xi)=\phi(2^{-j}\xi),\quad j\in\mathbb{N},\\ \phi_{0}(\xi)=1-\sum_{j=1}^{\infty}\phi_{j}(\xi). \end{cases} $$

Define the Littlewood-Paley (or dyadic) decomposition operators as

$$\Delta_{j}=\mathcal{F}^{-1}\phi_{j}\mathcal{F},\quad j\in\mathbb{N}\cup\{0\}. $$

Let $1\leq p, q \leq\infty, s\in\mathbb{R}$. For a tempered distribution *f*, we define the (inhomogeneous) Besov space $B_{p,q}^{s}$ as

$$B_{p,q}^{s}\bigl(\mathbb{R}^{n}\bigr):= \Biggl\{ f \in\mathcal{S}': \Vert f \Vert _{B_{p,q}^{s}(\mathbb{R}^{n})} = \Biggl(\sum _{j=0}^{\infty}2^{sjq} \Vert \Delta_{j}f \Vert _{L^{p}}^{q} \Biggr)^{\frac {1}{q}}< \infty \Biggr\} . $$

With the usual modification when $p, q=\infty$. Obviously, the *α*-decomposition is bigger than the uniform decomposition and thinner than the dyadic decomposition. This decomposition on frequency extends the dyadic and the uniform decomposition.

In order to prove the theorems, we also need some lemmas.

### Lemma 2.1

*Van der Corput lemma* ([15], *p*.334). *Let* *φ* *and* *ϕ* *be real valued smooth functions on the interval* $(a, b)$ *and* $k\in\mathbb{N}$. *If* $\vert \varphi^{(k)}(t) \vert \geq1$ *for all* $t\in(a, b)$ *and* (1) $k=1$, $\varphi^{\prime}(t)$ *is monotonic on* $(a, b)$, *or* (2) $k\geq2$, *then we have*

$$\biggl\vert \int_{a}^{b}e^{i\lambda\varphi(t)}\phi(t)\,dt \biggr\vert \leq c_{k}\lambda ^{-\frac{1}{k}} \biggl( \bigl\vert \phi(b) \bigr\vert + \int_{a}^{b} \bigl\vert \phi'(t) \bigr\vert \,dt \biggr). $$

### Lemma 2.2

(1) *If* $0\leq\alpha<1$, $1\leq p\leq\infty, s, s_{0} \in\mathbb{R}, k\in\mathbb{Z}^{n}$ *and*

$$\bigl\Vert \Box_{k}^{\alpha}(T_{\beta, \gamma} f) \bigr\Vert _{L^{p}}\preceq\langle k\rangle ^{\frac{s_{0}}{1-\alpha}} \Vert f \Vert _{L^{p}}, $$

*then*, *for any* $0< q\leq\infty$, $T_{\beta, \gamma} $ *is bounded from* $M_{p, q}^{s+s_{0}, \alpha}$ *to* $M_{p, q}^{s, \alpha}$.

(2) *If* $1\leq p\leq\infty, s, s_{0} \in\mathbb{R}, j\in\mathbb{Z}$ *and*

$$\bigl\Vert \Delta_{j}^{\alpha}(T_{\beta, \gamma} f) \bigr\Vert _{L^{p}}\preceq 2^{j s_{0} } \Vert f \Vert _{L^{p}}, $$

*then*, *for any* $0< q\leq\infty$, $T_{\beta, \gamma} $ *is bounded from* $B_{p, q}^{s+s_{0}, \alpha}$ *to* $B_{p, q}^{s, \alpha}$.

### Proof

(1) It suffices to show that

$$\bigl\Vert \Box_{k}^{\alpha}(T_{\beta, \gamma} f) \bigr\Vert _{L^{p}}\preceq\langle k\rangle ^{\frac{s_{0}}{1-\alpha}} \bigl\Vert \Box_{k}^{\alpha} f \bigr\Vert _{L^{p}}. $$

Denote $\Lambda(k)=\{j\in\mathbb{Z}^{n}:\eta_{k}^{\alpha}\cdot\eta_{j}^{\alpha }\neq0\}$. Then by the support condition (2.1), we have

$$\langle j\rangle\sim\langle k\rangle,\quad \mbox{if }j\in\Lambda(k) $$

and

$$\sharp\Lambda(k)\preceq1. $$

Recall that the choice of $\eta_{j}^{\alpha}$ satisfies $\sum_{j\in \mathbb{Z}^{n}} \eta_{j}^{\alpha} \equiv1$. By Minkowski’s inequality, we obtain

$$\begin{aligned} \bigl\Vert \Box_{k}^{\alpha}(T_{\beta, \gamma} f) \bigr\Vert _{L^{p}}&= \biggl\Vert \sum_{j\in\mathbb {Z}^{n}} \Box_{k}^{\alpha}\bigl[T_{\beta, \gamma} \bigl( \Box_{j}^{\alpha}f\bigr)\bigr] \biggr\Vert _{L^{p}} \leq\sum_{j\in\mathbb{Z}^{n}} \bigl\Vert \Box_{j}^{\alpha} \bigl[T_{\beta, \gamma} \bigl(\Box _{k}^{\alpha}f\bigr)\bigr] \bigr\Vert _{L^{p}} \\ &\preceq\sum_{j\in\Lambda(k)}\langle j\rangle^{\frac{s_{0}}{1-\alpha}} \bigl\Vert \Box_{k}^{\alpha}f \bigr\Vert _{L^{p}} \\ &\preceq\langle k\rangle^{\frac{s_{0}}{1-\alpha}} \bigl\Vert \Box_{k}^{\alpha} f \bigr\Vert _{L^{p}}. \end{aligned}$$

Thus, the first part of the lemma holds. The second part of this lemma is similar to the proof of the first part. Here we omit the details. □

## Main results and proofs

### Theorem 3.1

*Let* $\Gamma(t)= \vert t \vert ^{m}$ *or* $\Gamma(t)= \vert t \vert ^{m} \operatorname{sgn}(t) $. *If* $0\leq\alpha<1, \beta> 3\gamma>0, 1\leq p\leq\infty, 0<q\leq\infty$ *and* $s\in\mathbb{R}$, *then* $T_{\beta, \gamma}$ *is bounded from* $M_{p,q}^{s+\alpha,\alpha}$ *to* $M_{p,q}^{s,\alpha}$.

### Proof

By checking the following proof, we can only consider the operator

$$T_{\beta, \gamma}(f) (x, y)= \int_{0}^{1}f\bigl(x-t,y-t^{m}\bigr) \frac{e^{-2\pi i t^{-\beta}}}{t^{\gamma+1}}\,dt. $$

It is similar for $-1\leq t<0$. First, let us choose a $C^{\infty}$ function $\theta(t)$ with support in $[\frac{1}{2}, 2]$ on the real line satisfying

$$\sum_{j=0}^{\infty}\theta\bigl(2^{j}t \bigr)\equiv1. $$

Then we can decompose $T_{\beta, \gamma}$ as

3.1 $$ T_{\beta, \gamma}(f) (x, y)=\sum_{j=0}^{\infty} \int_{0}^{1}\theta \bigl(2^{j}t\bigr)f \bigl(x-t,y-t^{m}\bigr)\frac{e^{-2\pi i t^{-\beta}}}{t^{\gamma+1}}\,dt:=\sum _{j=0}^{\infty}T_{j}(f) (x, y). $$

Using the Fourier transformation, the operator $T_{j}$ can be written as

$$\widehat{T_{j}f}(\xi_{1}, \xi_{2})=m_{j}( \xi_{1}, \xi_{2})\hat{f}(\xi_{1}, \xi_{2}), $$

where

$$\begin{aligned} m_{j}(\xi_{1}, \xi_{2}) =& \int_{0}^{1} \theta\bigl(2^{j}t \bigr)e^{-2\pi i(\xi_{1}t+\xi _{2}t^{m})}\frac{e^{-2\pi i t^{-\beta}}}{t^{\gamma+1}}\,dt. \end{aligned}$$

Let $\Omega_{k, j}^{\alpha}$ be the kernel of $\Box_{k}^{\alpha}T_{j}$, so we have

$$\Box_{k}^{\alpha}T_{j}(f) (x)= \mathcal{F}^{-1}\bigl(\eta_{k}^{\alpha}(\xi )m_{j}(\xi)\bigr)\ast f(x)=\Omega_{k, j}^{\alpha}\ast f(x). $$

By the Young inequality, we obtain

$$\bigl\Vert \Box_{k}^{\alpha}T_{j}(f) \bigr\Vert _{L^{p}}\leq \bigl\Vert \Omega_{k, j}^{\alpha} \bigr\Vert _{L^{1}} \Vert f \Vert _{L^{p}}. $$

Let $\tilde{\eta} _{k}^{\alpha}(\zeta)=\eta_{k}^{\alpha}(\langle k\rangle^{\frac{\alpha }{1-\alpha}}\zeta+k\langle k\rangle^{\frac{\alpha}{1-\alpha}})$. Then $\operatorname{supp} \tilde{\eta} _{k}^{\alpha}(\zeta)\subset B(0,2r)$. By a simple substitution and the Fubini theorem, we get

$$\begin{aligned} \Omega_{k, j}^{\alpha}(x)={}& \int_{\mathbb{R}^{2}}e^{2\pi ix\cdot\xi}\eta _{k}^{\alpha}( \xi) \int_{0}^{1}\theta\bigl(2^{j}t \bigr)e^{-2\pi i(\xi_{1}t+\xi_{2}t^{m})}\frac {e^{-2\pi i t^{-\beta}}}{t^{\gamma+1}}\,dt\,d\xi_{1}\,d\xi_{2} \\ ={}& \int_{\mathbb{R}^{2}}\eta_{k}^{\alpha}(\xi) \int_{0}^{1}e^{-2\pi i[\xi _{1}(t-x_{1})+\xi_{2}(t^{m}-x_{2})]}e^{-2\pi i t^{-\beta}}\theta \bigl(2^{j}t\bigr)t^{-1-\gamma }\,dt\,d\xi_{1}\,d \xi_{2} \\ ={}&\langle k\rangle^{\frac{2\alpha}{1-\alpha}} \int_{\mathbb{R}^{2}}\tilde {\eta}_{k}^{\alpha}(\zeta) \int_{0}^{1}e^{-2\pi i[(\zeta_{1}\langle k\rangle ^{\frac{\alpha}{1-\alpha}}+k_{1}\langle k\rangle^{\frac{\alpha}{1-\alpha }})(t-x_{1})+(\zeta_{2}\langle k\rangle^{\frac{\alpha}{1-\alpha }}+k_{2}\langle k\rangle^{\frac{\alpha}{1-\alpha}})(t^{m}-x_{2})]} \\ &{}\times e^{-2\pi i t^{-\beta}}\theta\bigl(2^{j}t\bigr)t^{-1-\gamma}\,dt\,d \zeta_{1}\,d\zeta _{2} \\ ={}&\langle k\rangle^{\frac{2\alpha}{1-\alpha}}e^{2\pi i\langle k\rangle ^{\frac{\alpha}{1-\alpha}}k\cdot x} \int_{0}^{1}e^{-2\pi i(t^{-\beta }+k_{1}\langle k\rangle^{\frac{\alpha}{1-\alpha}}t+k_{2}\langle k\rangle ^{\frac{\alpha}{1-\alpha}}t^{m})}\theta \bigl(2^{j}t\bigr)t^{-1-\gamma} \\ &{}\times\hat{\tilde{\eta}}_{k}^{\alpha}\bigl(\langle k \rangle^{\frac{\alpha }{1-\alpha}}(t-x_{1}), \langle k\rangle^{\frac{\alpha}{1-\alpha}} \bigl(t^{m}-x_{2}\bigr)\bigr)\,dt. \end{aligned}$$

Let $\varphi(t)=-2\pi[t^{-\beta}+k_{1}\langle k\rangle^{\frac{\alpha }{1-\alpha}}t+k_{2}\langle k\rangle^{\frac{\alpha}{1-\alpha}}t^{m}]$, then

$$\begin{aligned} \Omega_{k, j}^{\alpha}(x)&= \langle k\rangle^{\frac{2\alpha}{1-\alpha }}e^{2\pi i\langle k\rangle^{\frac{\alpha}{1-\alpha}}k\cdot x} \int _{2^{-j-1}}^{2^{-j+1}}e^{i\varphi(t) }\theta \bigl(2^{j}t\bigr)t^{-1-\gamma}\hat {\tilde{\eta}}_{k}^{\alpha} \bigl(\langle k\rangle^{\frac{\alpha}{1-\alpha }}(t-x_{1}), \langle k \rangle^{\frac{\alpha}{1-\alpha}}\bigl(t^{m}-x_{2}\bigr)\bigr)\,dt \\ &:=\langle k\rangle^{\frac{2\alpha}{1-\alpha}}e^{2\pi i\langle k\rangle ^{\frac{\alpha}{1-\alpha}}k\cdot x}I_{j}. \end{aligned}$$

First, let us estimate $I_{j}$. We divide it into two cases.

Case 1. If $k_{2}\geq0$, then

$$\bigl\vert \varphi''(t) \bigr\vert =2\pi\bigl[ \beta(\beta+1)t^{-\beta-2}+m(m-1)k_{2}\langle k\rangle ^{\frac{\alpha}{1-\alpha}}t^{m-2}\bigr]\succeq t^{-\beta-2} \succeq2^{j(\beta+2)}. $$

By the Van der Corput lemma, we have

$$\begin{aligned} I_{j}\preceq{}&2^{-\frac{j(\beta+2)}{2}}2^{j(1+\gamma)} \bigl\vert \hat{ \tilde{\eta }}_{k}^{\alpha}\bigl(\langle k\rangle^{\frac{\alpha}{1-\alpha}} \bigl(2^{-j+1}-x_{1}\bigr), \langle k\rangle^{\frac{\alpha}{1-\alpha}} \bigl(2^{-jm+m}-x_{2}\bigr)\bigr) \bigr\vert \\ &{}+2^{-\frac{j(\beta+2)}{2}} \int_{2^{-j-1}}^{2^{-j+1}} \biggl\vert \frac {d}{dt} \bigl[\theta\bigl(2^{j}t\bigr)t^{-1-\gamma}\hat{\tilde{ \eta}}_{k}^{\alpha}\bigl(\langle k\rangle^{\frac{\alpha}{1-\alpha}}(t-x_{1}), \langle k\rangle^{\frac {\alpha}{1-\alpha}}\bigl(t^{m}-x_{2}\bigr)\bigr) \bigr] \biggr\vert \,dt \\ \preceq{}&2^{-\frac{j(\beta-2\gamma)}{2}} \bigl\vert \hat{\tilde{\eta}}_{k}^{\alpha } \bigl(\langle k\rangle^{\frac{\alpha}{1-\alpha}}\bigl(2^{-j+1}-x_{1}\bigr), \langle k\rangle^{\frac{\alpha}{1-\alpha}}\bigl(2^{-jm+m}-x_{2}\bigr)\bigr) \bigr\vert \\ &{}+2^{-\frac{j(\beta+2)}{2}} \int_{2^{-j-1}}^{2^{-j+1}}2^{j}\theta ' \bigl(2^{j}t\bigr)t^{-1-\gamma} \bigl\vert \hat{\tilde{ \eta}}_{k}^{\alpha}\bigl(\langle k\rangle ^{\frac{\alpha}{1-\alpha}}(t-x_{1}), \langle k\rangle^{\frac{\alpha }{1-\alpha}}\bigl(t^{m}-x_{2}\bigr)\bigr) \bigr\vert \,dt \\ &{}+2^{-\frac{j(\beta+2)}{2}} \int_{2^{-j}}^{2^{-j+1}}t^{-2-\gamma} \bigl\vert \hat { \tilde{\eta}}_{k}^{\alpha}\bigl(\langle k\rangle^{\frac{\alpha}{1-\alpha }}(t-x_{1}), \langle k\rangle^{\frac{\alpha}{1-\alpha}}\bigl(t^{m}-x_{2}\bigr)\bigr) \bigr\vert \,dt \\ &{}+2^{-\frac{j(\beta+2)}{2}}\langle k\rangle^{\frac{\alpha}{1-\alpha }} \int_{2^{-j}}^{2^{-j+1}}t^{-1-\gamma} \biggl\vert \frac{\partial\hat{\tilde {\eta}}_{k}^{\alpha}}{\partial x_{1}}\bigl(\langle k\rangle^{\frac{\alpha }{1-\alpha}}(t-x_{1}), \langle k\rangle^{\frac{\alpha}{1-\alpha }}\bigl(t^{m}-x_{2}\bigr)\bigr) \biggr\vert \,dt \\ &{}+2^{-\frac{j(\beta+2)}{2}}\langle k\rangle^{\frac{\alpha}{1-\alpha }} \int_{2^{-j}}^{2^{-j+1}}t^{-2-\gamma+m} \biggl\vert \frac{\partial\hat{\tilde {\eta}}_{k}^{\alpha}}{\partial x_{2}}\bigl(\langle k\rangle^{\frac{\alpha }{1-\alpha}}(t-x_{1}), \langle k\rangle^{\frac{\alpha}{1-\alpha }}\bigl(t^{m}-x_{2}\bigr)\bigr) \biggr\vert \,dt. \end{aligned}$$

Then, by a substitution and the Fubini theorem, we obtain

3.2 $$\begin{aligned} \bigl\Vert \Omega_{k, j}^{\alpha} \bigr\Vert _{L^{1}} \preceq{}&2^{-\frac{j(\beta-2\gamma)}{2}}\langle k\rangle^{\frac{2\alpha }{1-\alpha}} \int_{\mathbb{R}^{2}} \bigl\vert \hat{\tilde{\eta}}_{k}^{\alpha} \bigl(\langle k\rangle^{\frac{\alpha}{1-\alpha}}\bigl(2^{-j+1}-x_{1}\bigr), \langle k\rangle ^{\frac{\alpha}{1-\alpha}}\bigl(2^{-jm+m}-x_{2}\bigr) \bigr) \bigr\vert \,dx \\ &{}+2^{-\frac{j\beta}{2}}\langle k\rangle^{\frac{2\alpha}{1-\alpha}} \int _{\mathbb{R}^{2}} \int_{2^{-j-1}}^{2^{-j+1}}t^{-1-\gamma} \bigl\vert \hat{ \tilde{\eta }}_{k}^{\alpha}\bigl(\langle k\rangle^{\frac{\alpha}{1-\alpha}}(t-x_{1}), \langle k\rangle^{\frac{\alpha}{1-\alpha}}\bigl(t^{m}-x_{2}\bigr)\bigr) \bigr\vert \, dt\,dx \\ &{}+2^{-\frac{j(\beta+2)}{2}}\langle k\rangle^{\frac{2\alpha}{1-\alpha }} \int_{\mathbb{R}^{2}} \int_{2^{-j-1}}^{2^{-j+1}}t^{-2-\gamma} \bigl\vert \hat{ \tilde {\eta}}_{k}^{\alpha}\bigl(\langle k\rangle^{\frac{\alpha}{1-\alpha}}(t-x_{1}), \langle k\rangle^{\frac{\alpha}{1-\alpha}}\bigl(t^{m}-x_{2}\bigr)\bigr) \bigr\vert \, dt\,dx \\ &{}+2^{-\frac{j(\beta+2)}{2}}\langle k\rangle^{\frac{3\alpha}{1-\alpha }} \\ &{}\times\int_{\mathbb{R}^{2}} \int_{2^{-j-1}}^{2^{-j+1}}t^{-1-\gamma} \biggl\vert \frac {\partial\hat{\tilde{\eta}}_{k}^{\alpha}}{\partial x_{1}}\bigl(\langle k\rangle ^{\frac{\alpha}{1-\alpha}}(t-x_{1}), \langle k\rangle^{\frac{\alpha }{1-\alpha}}\bigl(t^{m}-x_{2}\bigr)\bigr) \biggr\vert \, dt\,dx \\ &{}+2^{-\frac{j(\beta+2)}{2}}\langle k\rangle^{\frac{3\alpha}{1-\alpha }} \\ &{}\times\int_{\mathbb{R}^{2}} \int_{2^{-j-1}}^{2^{-j+1}}t^{-2-\gamma+m} \biggl\vert \frac{\partial\hat{\tilde{\eta}}_{k}^{\alpha}}{\partial x_{2}}\bigl(\langle k\rangle^{\frac{\alpha}{1-\alpha}}(t-x_{1}), \langle k\rangle^{\frac {\alpha}{1-\alpha}}\bigl(t^{m}-x_{2}\bigr)\bigr) \biggr\vert \, dt\,dx \\ \preceq{}&2^{-\frac{j(\beta-2\gamma)}{2}} \bigl\Vert \hat{\tilde{\eta}}_{k}^{\alpha } \bigr\Vert _{L^{1}}+2^{-\frac{j\beta}{2}} \bigl\Vert \hat{\tilde{ \eta}}_{k}^{\alpha} \bigr\Vert _{L^{1}} \int_{2^{-j-1}}^{2^{-j+1}}t^{-1-\gamma}\,dt \\ &{}+2^{-\frac{j(\beta+2)}{2}} \bigl\Vert \hat{\tilde{\eta}}_{k}^{\alpha} \bigr\Vert _{L^{1}} \int _{2^{-j-1}}^{2^{-j+1}}t^{-2-\gamma}\,dt+2^{-\frac{j(\beta+2)}{2}} \langle k\rangle^{\frac{\alpha}{1-\alpha}} \biggl\Vert \frac{\partial\hat{\tilde{\eta }}_{k}^{\alpha}}{\partial x_{1}} \biggr\Vert _{L^{1}} \int _{2^{-j}}^{2^{-j+1}}t^{-1-\gamma}\,dt \\ &{}+2^{-\frac{j(\beta+2)}{2}}\langle k\rangle^{\frac{\alpha}{1-\alpha }} \biggl\Vert \frac{\partial\hat{\tilde{\eta}}_{k}^{\alpha}}{\partial x_{2}} \biggr\Vert _{L^{1}} \int_{2^{-j}}^{2^{-j+1}}t^{-2-\gamma+m}\,dt \\ \preceq{}&2^{-\frac{j(\beta-2\gamma)}{2}} \bigl\Vert \hat{\tilde{\eta}}_{k}^{\alpha } \bigr\Vert _{L^{1}}+2^{-\frac{j(\beta+2-2\gamma)}{2}}\langle k\rangle^{\frac {\alpha}{1-\alpha}} \biggl\Vert \frac{\partial\hat{\tilde{\eta}}_{k}^{\alpha }}{\partial x_{1}} \biggr\Vert _{L^{1}} +2^{-\frac{j(\beta+2m-2\gamma)}{2}} \langle k\rangle^{\frac{\alpha }{1-\alpha}} \biggl\Vert \frac{\partial\hat{\tilde{\eta}}_{k}^{\alpha }}{\partial x_{2}} \biggr\Vert _{L^{1}} \\ \preceq{}&2^{-\frac{j(\beta-2\gamma)}{2}}\langle k\rangle^{\frac{\alpha }{1-\alpha}}. \end{aligned}$$

Case 2. If $k_{2}<0$, then

$$\begin{aligned} \bigl\vert \varphi'''(t) \bigr\vert &= \bigl\vert -2\pi\bigl[-\beta(\beta+1) (\beta+2)t^{-\beta -3}+m(m-1) (m-3)k_{2}\langle k\rangle^{\frac{\alpha}{1-\alpha }}t^{m-3}\bigr] \bigr\vert \\ &\succeq t^{-\beta-3}\succeq2^{j(\beta+3)}. \end{aligned}$$

By the Van der Corput lemma, we have

$$\begin{aligned} I_{j}\preceq{}&2^{-\frac{j(\beta+3)}{3}}2^{j(1+\gamma)} \bigl\vert \hat{ \tilde {\eta}}_{k}^{\alpha}\bigl(\langle k\rangle^{\frac{\alpha}{1-\alpha }} \bigl(2^{-j+1}-x_{1}\bigr), \langle k\rangle^{\frac{\alpha}{1-\alpha }} \bigl(2^{-jm+m}-x_{2}\bigr)\bigr) \bigr\vert \\ &{}+2^{-\frac{j(\beta+3)}{3}} \int_{2^{-j-1}}^{2^{-j+1}} \biggl\vert \frac {d}{dt} \bigl[\theta\bigl(2^{j}t\bigr)t^{-1-\gamma}\hat{\tilde{ \eta}}_{k}^{\alpha}\bigl(\langle k\rangle^{\frac{\alpha}{1-\alpha}}(t-x_{1}), \langle k\rangle^{\frac {\alpha}{1-\alpha}}\bigl(t^{m}-x_{2}\bigr)\bigr) \bigr] \biggr\vert \,dt \\ \preceq{}&2^{-\frac{j(\beta-3\gamma)}{3}} \bigl\vert \hat{\tilde{\eta}}_{k}^{\alpha } \bigl(\langle k\rangle^{\frac{\alpha}{1-\alpha}}\bigl(2^{-j+1}-x_{1}\bigr), \langle k\rangle^{\frac{\alpha}{1-\alpha}}\bigl(2^{-jm+m}-x_{2}\bigr)\bigr) \bigr\vert \\ &{}+2^{-\frac{j(\beta+3)}{3}} \int_{2^{-j-1}}^{2^{-j+1}}t^{-1-\gamma }2^{j} \theta'\bigl(2^{j}t\bigr) \bigl\vert \hat{\tilde{ \eta}}_{k}^{\alpha}\bigl(\langle k\rangle ^{\frac{\alpha}{1-\alpha}}(t-x_{1}), \langle k\rangle^{\frac{\alpha }{1-\alpha}}\bigl(t^{m}-x_{2}\bigr)\bigr) \bigr\vert \,dt \\ &{}+2^{-\frac{j(\beta+3)}{3}} \int_{2^{-j-1}}^{2^{-j+1}}t^{-2-\gamma} \bigl\vert \hat{ \tilde{\eta}}_{k}^{\alpha}\bigl(\langle k\rangle^{\frac{\alpha}{1-\alpha }}(t-x_{1}), \langle k\rangle^{\frac{\alpha}{1-\alpha}}\bigl(t^{m}-x_{2}\bigr)\bigr) \bigr\vert \,dt \\ &{}+2^{-\frac{j(\beta+3)}{3}}\langle k\rangle^{\frac{\alpha}{1-\alpha }} \int_{2^{-j-1}}^{2^{-j+1}}t^{-1-\gamma} \biggl\vert \frac{\partial\hat{\tilde {\eta}}_{k}^{\alpha}}{\partial x_{1}}\bigl(\langle k\rangle^{\frac{\alpha }{1-\alpha}}(t-x_{1}), \langle k\rangle^{\frac{\alpha}{1-\alpha }}\bigl(t^{m}-x_{2}\bigr)\bigr) \biggr\vert \,dt \\ &{}+2^{-\frac{j(\beta+3)}{3}}\langle k\rangle^{\frac{\alpha}{1-\alpha }} \int_{2^{-j-1}}^{2^{-j+1}}t^{-2-\gamma+m} \biggl\vert \frac{\partial\hat {\tilde{\eta}}_{k}^{\alpha}}{\partial x_{2}}\bigl(\langle k\rangle^{\frac{\alpha }{1-\alpha}}(t-x_{1}), \langle k\rangle^{\frac{\alpha}{1-\alpha }}\bigl(t^{m}-x_{2}\bigr)\bigr) \biggr\vert \,dt. \end{aligned}$$

Thus, similar to (3.2), we get

3.3 $$\begin{aligned} \bigl\Vert \Omega_{k, j}^{\alpha} \bigr\Vert _{L^{1}}\preceq{}&2^{-\frac{j(\beta-3\gamma )}{3}} \bigl\Vert \hat{\tilde{ \eta}}_{k}^{\alpha} \bigr\Vert _{L^{1}} +2^{-\frac{j(\beta+3-3\gamma)}{3}}\langle k\rangle^{\frac{\alpha }{1-\alpha}} \biggl\Vert \frac{\partial\hat{\tilde{\eta}}_{k}^{\alpha }}{\partial x_{1}} \biggr\Vert _{L^{1}} \\ &{}+2^{-\frac{j(\beta+3m-3\gamma)}{3}}\langle k\rangle^{\frac{\alpha }{1-\alpha}} \biggl\Vert \frac{\partial\hat{\tilde{\eta}}_{k}^{\alpha }}{\partial x_{2}} \biggr\Vert _{L^{1}} \\ \preceq{}&2^{-\frac{j(\beta-3\gamma)}{3}}\langle k\rangle^{\frac{\alpha }{1-\alpha}}. \end{aligned}$$

Combining (3.2) and (3.3), we have

$$\bigl\Vert \Omega_{k, j}^{\alpha} \bigr\Vert _{L^{1}} \preceq2^{-\frac{j(\beta-3\gamma )}{3}}\langle k\rangle^{\frac{\alpha}{1-\alpha}}. $$

Therefore, noticing $\beta>3\gamma$ and combining with (3.1), we get

$$\bigl\Vert \Box_{k}^{\alpha}T_{\beta, \gamma}(f) \bigr\Vert _{L^{p}}\leq\sum_{j=0}^{\infty } \bigl\Vert \Box_{k}^{\alpha}T_{j}(f) \bigr\Vert _{L^{p}}\preceq\sum_{j=0}^{\infty}2^{-\frac {j(\beta-3\gamma)}{3}} \langle k\rangle^{\frac{\alpha}{1-\alpha}} \Vert f \Vert _{L^{p}}\preceq\langle k \rangle^{\frac{\alpha}{1-\alpha}} \Vert f \Vert _{L^{p}}. $$

So, by Lemma 2.2(1), we have

$$\bigl\Vert T_{\beta, \gamma}(f) \bigr\Vert _{M_{p,q}^{s,\alpha}}\preceq \Vert f \Vert _{M_{p,q}^{s+\alpha,\alpha}}. $$

We finished the proof of Theorem 3.1. □

### Theorem 3.2

*Let* $\Gamma(t)= \vert t \vert ^{m}$ *or* $\Gamma(t)= \vert t \vert ^{m} \operatorname{sgn}(t)$. *If* $\beta> \gamma >0, 1\leq p\leq\infty, 0< q\leq\infty$ *and* $s\in\mathbb{R}$, *then* $T_{\beta, \gamma}$ *is bounded from* $B_{p,q}^{s+1}$ *to* $B_{p,q}^{s}$.

### Proof

As the proof of Theorem 3.1, using the Fourier transformation, the operator $T_{\beta, \gamma}$ can be written as

$$\widehat{T_{\beta, \gamma}f}(\xi_{1}, \xi_{2})=m_{\beta, \gamma}( \xi_{1}, \xi _{2})\hat{f}(\xi_{1}, \xi_{2}), $$

where

$$m_{\beta, \gamma}(\xi_{1}, \xi_{2})= \int_{0}^{1} e^{-2\pi i(\xi_{1}t+\xi _{2}t^{m})}\frac{e^{-2\pi i t^{-\beta}}}{t^{\gamma+1}}\,dt. $$

Let $\Omega_{j, \beta, \gamma} $ be the kernel of $\Delta_{j} T_{\beta, \gamma}$, so we have

$$\Delta_{j} T_{\beta, \gamma}(f) (x)=\mathcal{F}^{-1}\bigl( \phi_{j} (\xi)m_{\beta , \gamma}(\xi)\bigr)\ast f(x)= \Omega_{j, \beta, \gamma} \ast f(x). $$

Using the Young inequality, we obtain

$$\bigl\Vert \Delta_{j} T_{\beta, \gamma}(f) \bigr\Vert _{L^{p}}\leq \Vert \Omega_{j, \beta, \gamma } \Vert _{L^{1}} \Vert f \Vert _{L^{p}}. $$

First, let us estimate $\Omega_{j, \beta, \gamma} $. By a substitution and integrating by parts, we have

$$\begin{aligned} \Omega_{j, \beta, \gamma} (x)={}& \int_{\mathbb{R}^{2}}e^{2\pi ix\cdot\xi }\phi_{j} (\xi) \int_{0}^{1}e^{-2\pi i(\xi_{1}t+\xi_{2}t^{m})}\frac{e^{-2\pi i t^{-\beta}}}{t^{\gamma+1}}\,dt\,d\xi \\ ={}& \int_{\mathbb{R}^{2}}\phi_{j} (\xi) \int_{0}^{1}e^{-2\pi i[\xi_{1}(t-x_{1})+\xi _{2}(t^{m}-x_{2})]}e^{-2\pi i t^{-\beta}}t^{-1-\gamma}\,dt\,d \xi_{1}\,d\xi_{2} \\ ={}&2^{2j} \int_{\mathbb{R}^{2}}\phi(\zeta) \int_{0}^{1}e^{-2\pi i[2^{j}\zeta _{1}(t-x_{1})+2^{j}\zeta_{2}(t^{m}-x_{2})]}e^{-2\pi i t^{-\beta}}t^{-1-\gamma }\,dt\,d \zeta_{1}\,d\zeta_{2} \\ ={}&\frac{2^{2j}}{2\pi\beta i} \int_{\mathbb{R}^{2}}\phi(\zeta)e^{-2\pi i[2^{j}\zeta_{1}(t-x_{1})+2^{j}\zeta_{2}(t^{m}-x_{2})]}e^{-2\pi i t^{-\beta}}t^{\beta -\gamma} \vert _{0}^{1}\,d\zeta \\ &{}-\frac{2^{2j}}{2\pi\beta i} \int_{\mathbb{R}^{2}}\phi(\zeta) \int_{0}^{1} e^{-2\pi it^{-\beta}}\,d\bigl[t^{\beta-\gamma}e^{-2\pi i[2^{j}\zeta _{1}(t-x_{1})+2^{j}\zeta_{2}(t^{m}-x_{2})]} \bigr]\,d\zeta \\ ={}&\frac{2^{2j}}{2\pi\beta i}\hat{\phi}\bigl(2^{j}(1-x_{1}), 2^{j}(1-x_{2})\bigr)e^{-2\pi i} \\ &{}-\frac{2^{2j}}{2\pi\beta i}(\beta-\gamma) \int_{\mathbb{R}^{2}}\phi (\zeta) \int_{0}^{1} e^{-2\pi it^{-\beta}}t^{\beta-\gamma-1}e^{-2\pi i[2^{j}\zeta_{1}(t-x_{1})+2^{j}\zeta_{2}(t^{m}-x_{2})]}\,dt\,d \zeta \\ &{}+\frac{2^{3j}}{\beta} \int_{\mathbb{R}^{2}}\phi(\zeta) \int_{0}^{1} e^{-2\pi it^{-\beta}}t^{\beta-\gamma}e^{-2\pi i[2^{j}\zeta_{1}(t-x_{1})+2^{j}\zeta _{2}(t^{m}-x_{2})]} \bigl(\zeta_{1}+\zeta_{2}mt^{m-1}\bigr)\,dt\,d\zeta. \end{aligned}$$

Then, using the Fubini theorem, we get

$$\begin{aligned} \bigl\vert \Omega_{j, \beta, \gamma} (x) \bigr\vert \preceq{}&2^{2j} \bigl\vert \hat{\phi}\bigl(2^{j}(1-x_{1}), 2^{j}(1-x_{2})\bigr) \bigr\vert \\ &{}+2^{2j} \biggl\vert \int_{0}^{1} e^{-2\pi it^{-\beta}}t^{\beta-\gamma-1}\hat {\phi}\bigl(2^{j}(t-x_{1}), 2^{j} \bigl(t^{m}-x_{2}\bigr)\bigr)\,dt \biggr\vert \\ &{}+2^{3j} \biggl\vert \int_{0}^{1} e^{-2\pi it^{-\beta}}t^{\beta-\gamma} \frac {\partial\hat{\phi}}{\partial x_{1}}\bigl(2^{j}(t-x_{1}), 2^{j} \bigl(t^{m}-x_{2}\bigr)\bigr)\,dt \biggr\vert \\ &{}+2^{3j} \biggl\vert \int_{0}^{1} e^{-2\pi it^{-\beta}}t^{\beta-\gamma+m-1} \frac {\partial\hat{\phi}}{\partial x_{2}}\bigl(2^{j}(t-x_{1}), 2^{j} \bigl(t^{m}-x_{2}\bigr)\bigr)\,dt \biggr\vert . \end{aligned}$$

Thus, using a substitution and the Minkowski inequality, it follows that

$$\begin{aligned} \Vert \Omega_{j, \beta, \gamma} \Vert _{L^{1}} \preceq{}&2^{2j} \int_{\mathbb{R}^{2}} \bigl\vert \hat{\phi}\bigl(2^{j}(1-x_{1}), 2^{j}(1-x_{2})\bigr) \bigr\vert \,dx \\ &{}+2^{2j} \int_{\mathbb{R}^{2}} \biggl\vert \int_{0}^{1} e^{-2\pi it^{-\beta }}t^{\beta-\gamma-1}\hat{ \phi}\bigl(2^{j}(t-x_{1}), 2^{j} \bigl(t^{m}-x_{2}\bigr)\bigr)\,dt \biggr\vert \,dx \\ &{}+2^{3j} \int_{\mathbb{R}^{2}} \biggl\vert \int_{0}^{1} e^{-2\pi it^{-\beta }}t^{\beta-\gamma} \frac{\partial\hat{\phi}}{\partial x_{1}}\bigl(2^{j}(t-x_{1}), 2^{j} \bigl(t^{m}-x_{2}\bigr)\bigr)\,dt \biggr\vert \,dx \\ &{}+2^{3j} \int_{\mathbb{R}^{2}} \biggl\vert \int_{0}^{1} e^{-2\pi it^{-\beta }}t^{\beta-\gamma+m-1} \frac{\partial\hat{\phi}}{\partial x_{2}}\bigl(2^{j}(t-x_{1}), 2^{j} \bigl(t^{m}-x_{2}\bigr)\bigr)\,dt \biggr\vert \,dx \\ \preceq{}& \Vert \hat{\phi} \Vert _{L^{1}}+2^{j} \biggl\Vert \frac{\partial\hat{\phi }}{\partial x_{1}} \biggr\Vert _{L^{1}}+2^{j} \biggl\Vert \frac{\partial\hat{\phi }}{\partial x_{2}} \biggr\Vert _{L^{1}} \\ \preceq{}&2^{j}. \end{aligned}$$

Therefore, we get

$$\bigl\Vert \Delta_{j}T_{\beta, \gamma}(f) \bigr\Vert _{L^{p}}\preceq2^{j} \Vert f \Vert _{L^{p}}. $$

Thus, following Lemma 2.2(2), we have

$$\bigl\Vert T_{\beta, \gamma}(f) \bigr\Vert _{B_{p,q}^{s}}\preceq \Vert f \Vert _{B_{p,q}^{s+1}}. $$

We finished the proof of Theorem 3.2. □

### Theorem 3.3

*If* $0\leq\alpha\leq1$, $\beta> (n+1)\gamma , 1\leq p\leq\infty, 0< q\leq\infty$ *and* $s\in\mathbb{R}$, *then* $T_{n, \beta, \gamma}$ *is bounded from* $M_{p,q}^{s+\alpha,\alpha}$ *to* $M_{p,q}^{s,\alpha}$.

### Proof

When $\alpha=1$, it is similar to the proof of Theorem 3.2. Thus, we only prove the case of $0\leq\alpha<1$. Checking the proof of this theorem, we only need to show the case, $0< t\leq1$. When $-1\leq t<0$, the proof is similar. For convenience, we rewrite the operator as follows:

$$T_{n, \beta, \gamma}f(x)= \int_{0}^{1}f\bigl(x-\Gamma_{\theta}(t) \bigr)\frac {e^{-2\pi i t^{-\beta}}}{t^{1+\gamma}}\,dt. $$

As in Theorem 3.1, choose a $C^{\infty}$ function $\Psi(t)$ with support in $[\frac{1}{2}, 2]$ on the real line satisfying

$$\sum_{j=0}^{\infty}\Psi\bigl(2^{j}t \bigr)\equiv1. $$

Then we can decompose $T_{n, \beta, \gamma}$ as

$$T_{n, \beta, \gamma}(f) (x)=\sum_{j=0}^{\infty} \int_{0}^{1}\Psi \bigl(2^{j}t\bigr)f \bigl(x-\Gamma_{\theta}(t)\bigr)\frac{e^{-2\pi i t^{-\beta}}}{t^{\gamma +1}}\,dt:=\sum _{j=0}^{\infty}T_{n,j}(f) (x). $$

Using the Fourier transformation, the operator $T_{n, j}$ can be written as

$$\widehat{T_{n, j}f}(\xi)=m_{n, j}(\xi)\hat{f}(\xi), $$

where

$$m_{n, j}(\xi)= \int_{0}^{1} \Psi\bigl(2^{j}t \bigr)e^{-2\pi i(\Gamma_{\theta}(t)\cdot\xi +t^{-\beta})}\frac{1}{t^{\gamma+1}}\,dt. $$

Therefore, we have

$$\Box_{k}^{\alpha}T_{n, j}(f) (x)= \mathcal{F}^{-1}\bigl(\eta_{k}^{\alpha}m_{n, j} \bigr)\ast f(x). $$

By the Young inequality, we get

$$\bigl\Vert \Box_{k}^{\alpha}T_{n, j}(f) \bigr\Vert _{L^{p}}\leq \bigl\Vert \mathcal{F}^{-1}\bigl(\eta _{k}^{\alpha}(\cdot)m_{n, j}(\cdot)\bigr) \bigr\Vert _{L^{1}} \Vert f \Vert _{L^{p}}. $$

Now, we estimate $\Vert \mathcal{F}^{-1}(\eta_{k}^{\alpha}(\cdot)m_{n, j}(\cdot)) \Vert _{L^{1}}$. By scaling, we can assume $\theta=(1, 1, \ldots, 1)$ in the definition of $\Gamma_{\theta}(t)$. Let $\tilde{\eta} _{k}^{\alpha}(\zeta)=\eta_{k}^{\alpha}(\langle k\rangle^{\frac{\alpha }{1-\alpha}}\zeta+k\langle k\rangle^{\frac{\alpha}{1-\alpha}})$. By a simple substitution and the Fubini theorem, we obtain

$$\begin{aligned} &\mathcal{F}^{-1}\bigl(\eta_{k}^{\alpha}m_{n, j} \bigr) (x) \\ &\quad= \int_{\mathbb{R}^{n}}e^{2\pi ix\cdot\xi}\eta_{k}^{\alpha}( \xi) \int _{0}^{1}\Psi\bigl(2^{j}t \bigr)e^{-2\pi i (\Gamma_{\theta}(t)\cdot\xi+t^{-\beta })}t^{-1-\gamma}\,dt\,d\xi \\ &\quad= \int_{0}^{1}e^{-2\pi it^{-\beta}}t^{-1-\gamma}\Psi \bigl(2^{j}t\bigr) \int_{\mathbb {R}^{n}} \eta_{k}^{\alpha}( \xi)e^{-2\pi i[(t^{p_{1}}-x_{1})\xi _{1}+(t^{p_{2}}-x_{2})\xi_{2}+\cdots+(t^{p_{n}}-x_{n})\xi_{n}]}\,d\xi \,dt \\ &\quad =e^{\sum_{l=1}^{n}2\pi ix_{l}k_{l}\langle k\rangle^{\frac{\alpha}{1-\alpha }}}\langle k\rangle^{\frac{n\alpha}{1-\alpha}} \int_{0}^{1}e^{-2\pi i[t^{-\beta}+\sum_{l=1}^{n}k_{l} \langle k\rangle^{\frac{\alpha}{1-\alpha }}t^{p_{l}}]}t^{-1-\gamma}\Psi \bigl(2^{j}t\bigr) \\ &\qquad{}\times \int_{\mathbb{R}^{n}}\tilde{\eta}_{k}^{\alpha}( \zeta)e^{-2\pi i\sum _{l=1}^{n}(t^{p_{l}}-x_{l})\zeta_{l}\langle k\rangle^{\frac{\alpha}{1-\alpha }}}\,d\zeta \,dt \\ &\quad=e^{\sum_{l=1}^{n}2\pi ix_{l}k_{l}\langle k\rangle^{\frac{\alpha}{1-\alpha }}}\langle k\rangle^{\frac{n\alpha}{1-\alpha}} \int _{2^{-j-1}}^{2^{-j+1}}e^{i \phi(t)}t^{-1-\gamma}\Psi \bigl(2^{j}t\bigr) \\ &\qquad{}\times\hat{\tilde{\eta}}_{k}^{\alpha}\bigl(\langle k \rangle^{\frac{\alpha }{1-\alpha}}\bigl(t^{p_{1}}-x_{1}\bigr), \langle k \rangle^{\frac{\alpha}{1-\alpha }}\bigl(t^{p_{2}}-x_{2}\bigr), \ldots, \langle k\rangle^{\frac{\alpha}{1-\alpha }}\bigl(t^{p_{n}}-x_{n}\bigr) \bigr)\,dt. \end{aligned}$$

Denote $\phi(t)=-2\pi[t^{-\beta}+\sum_{l=1}^{n}k_{l} \langle k\rangle ^{\frac{\alpha}{1-\alpha}}t^{p_{l}}]$. By a normal computation, we get

$$\phi^{(m)}(t)=-2\pi(-1)^{m} \bigl[\beta(\beta+1)\cdots( \beta+m-1)t^{-\beta -m}+(-1)^{m}A_{m}^{0}(t)t^{-m} \bigr], $$

where

$$A_{m}^{0}(t)=\sum_{l=1}^{n}k_{l} \langle k\rangle^{\frac{\alpha}{1-\alpha }}p_{l}(p_{l}-1) \cdots(p_{l}-m+1)t^{p_{l}}. $$

Set $K_{m}=\{t: (-1)^{m}A_{m}^{0}(t)\geq0 \}$. Checking the proof in [3], when $t\in K_{m}$, we get

$$\bigl\vert \phi^{(m)}(t) \bigr\vert \geq C_{\beta, m}t^{-\beta-m}. $$

Then, using the ideas in [3] and following the proofs in Theorem 3.1, we obtain Theorem 3.3. Here we omit the details. □

### Remark 3.4

In Theorems 3.1 and 3.3, if we take $\alpha=0$, we will get the sufficient results in [4]. Unlike [4], we use the discrete definition of modulation spaces. So, our method is different from [4]. Unfortunately, if $0<\alpha\leq1$, for the scaling property of the decompositions(see Section 2 for details), we will lose the regularity of the space by our method. Maybe we need some new ideas to overcome this limitation.

### Remark 3.5

In [16], the authors mention the fact that the *α*-modulation space cannot be obtained by interpolation between modulation spaces $\alpha= 0$ and Besov spaces $\alpha= 1$. Thus, it shows that our proofs for $0 < \alpha< 1$ in Theorems 3.1 and 3.3 are meaningful.

### Remark 3.6

We can extend this result to the well-curved *γ* in $\mathbb{R}^{n}$. Let $\gamma(t)$ be a smooth mapping such that $\gamma(0)=0$ and

$$\frac{d^{k}\gamma(t)}{dt^{k}}\Big| _{t=0}, \quad k=1, 2, \ldots $$

$\operatorname{span} \mathbb{R}^{n}$ (smooth mappings of finite type in a small neighborhood of the origin). Then we call $\gamma(t)$ well curved. See [17, 18] for details. According to [17] (Proposition 3.1), to every smooth well-curved $\gamma(t)$ there exists a constant nonsingular matrix *M* such that $\tilde{\gamma}(t)=M\gamma(t)$ is of standard type; that is, approximately homogeneous, taking the form

$$\gamma_{k}(t)=\frac{t^{a_{k}}}{a_{k}!}+\mbox{higher order terms} $$

for $k=1, 2, \ldots, n$ with $1\leq a_{1}< a_{2} < \cdots<a_{n}, \tilde {\gamma}(t)=(\gamma_{1}(t), \gamma_{2}(t), \ldots, \gamma_{n}(t))$. Following the ideas in [17], combining with Theorem 3.3, we can get

### Theorem 3.7

*Let* $\Gamma_{\theta}(t)$ *be well*-*curved*. *If* $0\leq\alpha \leq1$, $\beta> (n+1)\gamma, 1\leq p\leq\infty, 0< q\leq\infty$ *and* $s\in\mathbb{R}$, *then* $T_{n, \beta, \gamma}$ *is bounded from* $M_{p,q}^{s+\alpha,\alpha}$ *to* $M_{p,q}^{s,\alpha}$.

## Conclusions

In this paper, using the equivalent discrete definition of *α*-modulation spaces, combining the Fourier transform and Van der Corput lemma, we obtained the strongly singular integrals along homogeneous curves are bounded on the *α*-modulation spaces for all $0\leq \alpha\leq1$. Our results extend the main results in [4]. Our method is also different from [4].
